# Pre-anaesthesia Telephone Consultation: A Safe Alternative for Anaesthesia Assessment in Case of Repeated Low or Intermediate Risk Surgeries: A Prospective Cohort Study

**DOI:** 10.4274/TJAR.2022.221079

**Published:** 2023-06-16

**Authors:** Charles-Herve Vacheron, Clemence Ferrier, Estelle Morau, Alexandre Theissen, Vincent Piriou, Pierre Yves Carry, Arnaud Friggeri

**Affiliations:** 1 Department of Anaesthesia Resuscitation, Centre Hospitalier Lyon Sud, Hospices Civils de Lyon, Lyon, France; 2 CIRI-International Center for Infectiology Research (Team PHE3ID), Univ Lyon, University Claude Bernard Lyon 1, Lyon, France; 3 Department of Anaesthesia Resuscitation, University Claude Bernard Lyon 1 Faculty of Medicine Lyon Sud, Lyon, France; 4 Department of Anaesthesia Resuscitation, Centre Hospitalier de Mayotte, Mamoudzou, France; 5 Department Anaesthesia Resuscitation, Douleur Urgence CHU Carémeau, Nîmes, France; 6 Department of Anaesthesia Resuscitation, Centre Hospitalier Princesse Grâce, Monaco, Monako; 7 Department of Anaesthesia Resuscitation, Centre Hospitalier Lyon Sud, Hospices Civils de Lyon, Pierre-Bénite, France

**Keywords:** Anaesthesia, consultation, telemedicine

## Abstract

**Objective:**

Telemedicine has widely expanded during the coronavirus disease-2019 pandemic. Our objective was to evaluate the feasibility, safety, effectiveness, and satisfaction of pre-anaesthesia telephone consultation (PATC).

**Methods:**

From December 2015 to October 2016, a prospective survey was administered to anaesthesiologists, nurse anaesthetists, and patients of the ambulatory and maxillofacial departments. Patients having a pre-anaesthesia consultation (PAC) within the previous year in the department, whose health state was considered stable, and for whom the surgical procedure was related to the previous one, were eligible for PATC. Three questionnaires concerning the pre- (Q1), per- (Q2), and postoperative (Q3) periods were answered by the patient, the anaesthesiologist, and the anaesthesiologist nurse to evaluate the feasibility and satisfaction of the PATC. We collected the cancelation rate and any incident occurring during the surgery.

**Results:**

Over the study period, 210 patients were included. The response rate was 200/210 (95.2%) for Q1, 108/208 (51.9%) for Q2 and 146/208 (70.2%) for Q3. PATC was performed in a median (IQR) of 13 (7-20) days before the procedure. Patients answered directly in 73% of cases without the need for recall. During surgery, 4 incidents occurred and none were attributable to PATC. Patient satisfaction was 93.3% and 85.8% of them preferred PATC to conventional PAC. The kilometric saving was 74 (30-196) km per PATC.

**Conclusion:**

Both patients and professionals were satisfied with PATC, which did not impact safety. On the selected patients, PATC brings many practical benefits and increases organizational flexibility.

**Main Points**

• Pre-operative anaesthesia consultation allows a better patient safety.

• The pre-anaesthesia telephone consultation (PTAC) replacing physical consultation, allowed a good satisfaction both for healthcare professionals, and for the patient.

• The PTAC is a safe and reliable alternative in case of low or intermediate surgery.

## Introduction

Telemedicine combines the benefit of an equitable, affordable, and accessible way to healthcare resources.^1,2^ While telemedicine has widely expanded during the coronavirus disease-2019 pandemic, it has currently been inadequately assessed concerning its safety and efficiency. Carrying out a pre-anaesthetic assessment optimizes the quality of perioperative care and anaesthetic safety while contributing to the economic efficiency at the hospital level.^3,4^ In France, pre-anaesthesia consultation (PAC) has been legally mandatory since 1993 for every patient before anaesthesia, including isolated locoregional anaesthesia (LRA). The PAC aimed to evaluate the clinical condition of the patient, prepare the patient for surgery, and inform the patient about the modality of anaesthesia and analgesia in the perioperative period. In collaboration with the operator, the patient is able to give informed consent to the planned procedure and choose his modality of anaesthesia (LRA, general anaesthesia). Prior to each procedure, this consultation must be carried out by an anaesthesiologist, at least 48 hours before the intervention.^5^

Briefly, the PAC consists of a complete medical consultation: searches for medical, surgical, and anaesthetic history, current treatments, and history of allergy. The anaesthesiologist performs a physical examination, mainly to evaluate the predictable difficulty of ventilation or intubation. Moreover, the anaesthesiologist plans the potential discontinuation of medications interfering with anaesthesia and surgery, including antiplatelet, anticoagulant, or some antihypertensive drugs. Then, just before surgery, the anaesthesiologist performs a preoperative assessment based on this consultation.

Since 2014, pre-anaesthesia telephone consultation (PATC) has been performed as an innovative experiment in selected patients after governmental authorization. In 2017, the commission on risk assessment and risk management of the French society of anesthesiology [CAMR, Société Française d’Anaesthésie Réanimation, (SFAR)] defined the modalities for PATC, mainly to avoid patients’ traveling in the case of repeated surgeries (surgical procedure related to the previous one).^6^ However, there has been no evaluation of this practice in this situation in France.

We hypothesize that PTAC is a feasible and safe alternative to physical PAC. We therefore conducted this study to evaluate PATC as an alternative to traditional consultations for known patients in cases of low- or intermediate- risk surgeries following a previous surgical procedure with a previous pre-anaesthesia consultation. Our primary outcome was to estimate the surgery cancelation rate associated with PATC, and the secondary outcome included the evaluation of the quality, safety, feasibility, and satisfaction of PTAC.

## Methods

### Study Setting

A prospective single-center survey study was conducted between December 16^th^ 2015 and October 16^th^ 2016 (10 month). This survey focused on patients undergoing maxillofacial or outpatient surgery in a French University hospital. Consent was obtained from all participants. The study was conducted in accordance with the ethical standards laid down in the 1964 Declaration of Helsinki and its later amendments. This study was approved by the Hospices Civiles de Lyon Committee (approval no: 19-139, date; 2015).

### PATC Criteria

Inclusion criteria for the patient to be eligible for PATC were:

- ASA scores 1, 2 or 3.

- Stable health state.

- Previous physical PAC within the previous year for surgical or interventional procedure related to the consultation.

- No pre-operative blood testing needed.

- Agreements from the patients, surgeons, and anaesthesiologists.

The patients were informed that they could withdraw their consent anytime throughout the study period. The anaesthesiologist in charge could also decide to redirect during the PATC the patient to a conventional consultation.

### Survey

Data were collected using 3 questionnaires drawn up by the anaesthesia team according to the 2013 guidelines of the French Health Authority.^7^ They included closed dichotomous and multiple-choice questions, satisfaction or judgment scales, Likert scale-type or numeric and semantic closed-order scales, and additional open-ended questions (Appendix).

The first questionnaire (Q1 - supplementary data A) was divided into two parts. The first part was intended for the patient to assess his satisfaction. With 5 questions, it explored satisfaction regarding the technical modalities, quality and safety, and practical benefits provided by the PATC. The second part was responded to by the anaesthesiologist conducting the consultation. The physician answered eight questions concerning the patient’s epidemiological data, information concerning PATC technical modalities (number of calls, consultation time frame, and connection quality), practical benefits of this type of consultation, and the anaesthesiologist’s overall satisfaction.

The second questionnaire (Q2 - supplementary data B) was completed by the nurse anaesthetists. It aimed at collecting information during the perioperative period, with 11 questions regarding the efficiency, safety, and satisfaction regarding PATC. We also collected incidents such as intervention cancelation or postponement. Finally, in the third questionnaire (Q3 - supplementary data C) completed in the postoperative period, five questions were asked to the patient regarding his final satisfaction.

### Objective

We estimated the surgery cancelation rate associated with PATC.

### Secondary objectives were;

- The evaluation of the quality and safety of PATC, especially regarding the rate of incident attributable to PATC.

- The evaluation of the organizational and technical feasibility of the PATC.

- The evaluation of patient and anaesthesiologist satisfaction and benefit from the PATC.

### Statistical Analysis

Descriptive statistics were expressed as the mean ± standard deviation or median (Q1-Q3) according to the normality of their distributions. Statistical analysis was performed using R software V 3.6.3.

## Results

Over the study period, 210 patients were included (48% of the PATC performed in the different departments of the hospital during the study period). A total of 454 questionnaires was analysed. The response rate was 200/210 (95%) for Q1 (intended for anaesthesiologists and their patients after PATC), 108/208 (51%) for Q2 (intended for nurse anaesthetists on the day of the intervention) and 146/208 (70.2%) for Q3 (intended for patients in the postoperative period). Patients were 42 ± 21 years old, 198 (94%) were ASA 1 or 2, 172 (83%) had an outpatient surgery, 188 (90%) had a general anaesthesia (Tables 1, 2). PATC were performed in a median of 193 (84-313) days after the previous in-person PAC.

Among the 210 procedures planned after PATC, 2 (<1%) were cancelled because one for non-respect of fasting and the other for non-withdrawal of antithrombotic treatment, which was introduced after PATC. Apart from cancellations, 4 incidents were reported, 3 related to difficult airway predictors and 1 to unreported allergy. These incidents did not require a modification of the anaesthesia protocol.

### Feasibility and Effectiveness

Median delay between PATC and surgical procedure was 13 (7-20) days, and the mean duration of PATC was 12 ± 4 minutes. The patient response was obtained upon first call in 75.0% of cases and a second call was necessary in 19% of cases. Anaesthesiologists considered the setting as optimal in 92.7% of PATC performed. Otherwise, the reasons given were poor technical conditions (n = 4), poor preparation of the patient for the consultation (n = 4), communication problems (n = 2), patient anxiety (n = 2), or patient incivility (n = 2).

### Benefits and Satisfaction

The main advantage reported by patients was the possibility of performing the consultation outside the hospital, thus avoiding the difficulties related to organization of professional activities.

The median distance between patients’ home and hospital was 37 (15-98) km. Geographic distance was not the only concern raised by patients: traffic jams, parking problems, lack of vehicle, limited access to public transportation, need of a third party, or physical disability were also reported as advantages. The other benefits mentioned included the possibility of maintaining the surgery date despite the overloaded anaesthesia consultations and the time saved.

**Figure 1:**
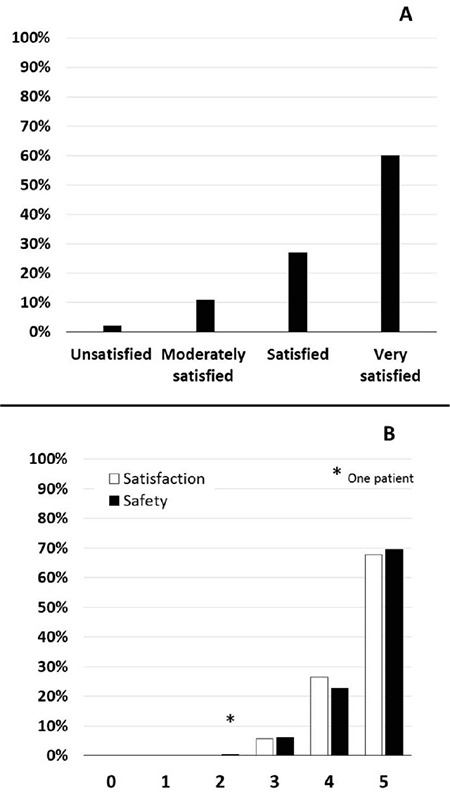
PATC satisfaction and safety evaluation by patients (A) and satisfaction evaluation by anaesthesiologists (B).

Among the anaesthesiologists, 87% were satisfied or very satisfied (Figure 1A) but 22% questioned the validity of PATC due to concerns regarding clinical examination (4%), patient reliability (2%), patient understanding (2%), or medical history (1%). According to 94% of the nurse anaesthetists, PATC did not alter management in the operating room. Exhaustivity of PATC was reported in 96% of cases, adequate patient information in 98%, and satisfaction from patients in 97% of cases.

Finally, patient satisfaction and safety scores were high (Figure 1B), with a 4-5 score in 93% and 92.3% respectively. Benefits provided by PATC were reported in 98% of cases and related to distance, occupation and childcare in 70%, 38% and 3% of cases, respectively.

Following the intervention, 85% of patients felt safe using this type of consultation, 94% found PATC modalities effective, 96% reported practical benefits of PATC for which 76% was related to travel, 36% to occupation, and 6% to childcare, and 98% considered that all their questions were answered. Overall, 85.8% of the surveyed patients preferred PATC over conventional in-person consultation.

## Discussion

PATC intends to assist and simplify consultation without degrading anaesthetic safety. In this study, the inclusion criteria entailed that PATC was performed in rather young and active patients with good medical conditions. In this context, the cancelation rate was low and not related to an error performed during the consultation. This is lower than the cancelation rates for elective surgery reported in the literature, ranging between 5% and 40%.^8,9,10,11^

Although several studies have evaluated pre-anaesthesia patient health status by telephone relying on a precise checklist, PATC herein consisted in a true consultation except for the lack of clinical examination.^9,11,12^ In the French medical setting, in-person PAC is considered a legal gold standard, and any modification of the latter is a priori considered harmful for the patient and risky for the anaesthesiologist. Indeed, in France, the anaesthesiologist is in charge of all the medical and drug management related issues associated with surgery in the pre-, per-, and postoperative period. This explains the importance given to PAC by French anaesthesiologists. Importantly, the anaesthesiologist’s professional satisfaction with PATC in this study seemed excellent. Following the severe acute respiratory syndrome coronavirus-2 outbreak, to avoid any contamination, telemedicine has spread widely in France, also in the preanaesthesia area.

PATC was performed in obstetrical setting and authors report a lack of information in 10% of cases and that this constituted a loss of opportunity in 1.5% of cases.^13^ Our results indicated a lower rate, probably due to the prior evaluation of the patient and his medical records.

Others perform telehealth consultation that differs from PATC in that it uses a videoconference system. Telehealth consultation mimes face-to-face consultation in allowing physical examination (i.e., upper airway, venous access…) and improving patient-physician interaction.^14,15^ Telehealth consultation is promising but was not available at the time of our study. In the future, our system could be improved by video conference tools.

Telemedicine for pre-anaesthesia assessment was also tested in Canada and USA, two very large countries with low medical density areas.^16,17^ Teleconsultation facilitates care access in geographically remote areas or in those deficient in care offer. It avoids patients traveling and thus reduces transport costs when the patient is distant from the care centre or in case of mobility impairment.^18,19^ In previous studies, pre-anaesthesia teleconsultation was reported as safe and satisfying.^20,21^

Although the airway evaluation is a concern for anaesthesiologists, the observation of difficult airway predictors on the day of surgery was not associated with the procedure cancellation. Moreover, no serious adverse events were associated with the PATC procedure. Nevertheless, an ultimate check of the patients condition (“pre-anaesthesia visit”) just before surgery is mandatory in France. During this visit, risk of difficult airway management can be assessed although, to date, airway evaluation scores have a low sensitivity.^22^

Regarding practical modalities, the results here showed that it is possible to perform PATC under good conditions within a reasonable time frame before surgery, highlighting more flexibility than conventional PAC for both patients and practitioners. This method can avoid the operative cancelation for patients who could not undergo PAC before the intervention due to lack of consultation availability. Moreover, teleconsultation could be implemented as a homeworking solution to save both the patient and anaesthesiologist a round trip to the hospital, thus improving their quality of life. Patient satisfaction was also excellent. From the patient perspective, PATC was considered effective and safe. The level of satisfaction observed here was comparable to those reported in the literature using telephone or video conference.^9,10,20,22,23^

In the case of communication impairment (deafness, tracheostomy, foreign language), the use of a third-party should help conduct PATC in good conditions. However, this raises the issue of patient confidentiality; the third-party role must be defined beforehand and should not be a hindrance to its realisation.

The most frequently highlighted benefits were travel and time savings. PATC brings a real benefit to people with limited autonomy and to the working population. Furthermore, the economic impact of PATC cannon be reduced solely by travel costs. Reduced costs concerning days off or childcare associated with increased flexibility in the management of medical time and surgery schedulings should also be considered.

Finally, the ecological benefit for the economy of road travel must be regarded in the global climatic crisis toward which we are going.

### Study Limitation

The interpretation of our results needs to be considered regarding the usual precautions of a small, monocentric study. Indeed, the study is underpowered to detect a difference in very rare unusual events, and the PTAC might not fit in surgery requiring a lot of preoperative evaluation.

## Conclusion

PATC may be an alternative to physical PAC in cases of repeated surgery and outpatient surgery. Despite being beneficial, PATC needs to be evaluated regarding the patient safety and cancelation rate of operations in severely ill patients and compared to a classical physical consultation.

## Figures and Tables

**Table 1:**
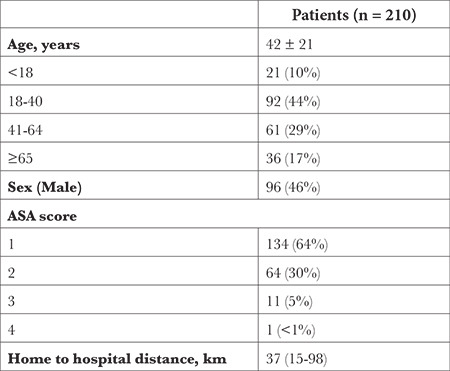
Patient Characteristics

**Table 2:**
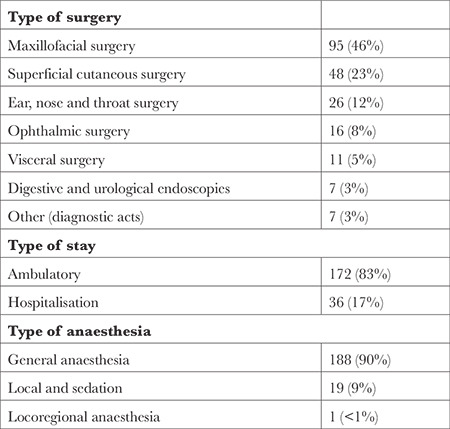
Procedure Characteristics
